# Genetic and stem cell insights into the pathogenesis of endometriosis: A comprehensive review

**DOI:** 10.14440/jbm.0148

**Published:** 2025-10-02

**Authors:** Sheena Mariam Thomas, Ramakrishnan Veerabathiran

**Affiliations:** Human Cytogenetics and Genomics Laboratory, Faculty of Allied Health Sciences, Chettinad Hospital and Research Institute, Chettinad Academy of Research and Education, Kelambakkam 60313, Tamil Nadu, India

**Keywords:** Endometriosis, Pathophysiology, Genetic basis, Stem cells, Infertility

## Abstract

**Background:**

Endometriosis represents a predominant gynecological disease that globally impacts 10% of women in their reproductive years, often leading to pelvic pain, infertility, and other complications.

**Objective:**

This review intended to impart a comprehensive understanding of the pathophysiology, inherent genetic susceptibility, and the role of stem cells in the progression of endometriosis. It explores the diagnostic challenges posed by the diverse presentation of lesions and the involvement of genetic factors, including genes related to inflammation, immune response, steroidogenesis, neo-angiogenesis, and DNA repair. In addition, the role of adult stem cells, particularly from bone marrow and the endometrium is highlighted as an important aspect of disease progression. The review also examined how environmental factors, including early menarche, heavy menstrual flow, and Müllerian anomalies, contribute to endometriosis development. The impact on fertility is discussed concerning pelvic anatomical distortions, which affect egg release, sperm motility, and embryo transit. Furthermore, the review addressed complications, such as chronic pelvic pain, dysmenorrhea, dyspareunia, and potential obstetric issues, including an increased risk of certain cancers. Finally, it emphasized the need for improved diagnostic techniques and targeted therapies, focusing on early detection, innovative treatments, and fertility preservation.

**Conclusion:**

Advancements in genomic and molecular research are crucial to understanding the genetic basis of endometriosis and ultimately enhance the quality of life for those affected.

## 1. Introduction

Endometriosis is a raging gynecological disease impacting approximately 10% of women in their reproductive years, as exemplified by the incidence of endometrium-like tissues found outside the uterine cavity. This condition is associated with pelvic pain and an increased likelihood of infertility.^1^ It is a common disease that presents a significant public health problem and is also a leading cause of hysterectomy.^2^ Endometriosis is a kind of chronic inflammatory disease, an estrogen-dependent condition causing such symptoms as dyspareunia, dysmenorrhea, dysuria, chronic fatigue, dyschezia, and chronic pelvic pain.^3^ The pathogenesis of endometriosis is complicated and multifactorial, and is proposed to involve several mechanisms, including retrograde menstruation, coelomic metaplasia, immune dysfunction, environmental influences, and, notably, genetic predisposition. Studies suggested that while retrograde menstruation occurs in over 90% of menstruating women, only 6–10% develop endometriosis, indicating that genetic and molecular factors play a critical role in disease susceptibility and progression.^4^

Among the genetic factors, chromosomal region 10q26 has been found to be significantly associated with endometriosis, particularly in familial cases. Recent genome-wide association studies (GWAS) have also identified other underlying loci, such as 7p15.2, further supporting the notion that a hereditary component is implicated in the endometriosis pathophysiology.^5^

Epidemiological data estimated that annual incidence rates stood somewhere between 0.112% and 0.72%.^5^ Diagnosis is often delayed due to the non-specific nature of symptoms and the need for invasive procedures for confirmation. Laparoscopic evaluation remains the benchmark for diagnosis, allowing for direct visualization of ectopic endometrial lesions.^6^ These lesions vary widely in morphology, size, and color, and are typically categorized into three types: Superficial peritoneal lesions, endometriomas, and deep-infiltrating nodules.^7^ Histopathological confirmation requires identifying key features, such as endometrial stroma, glands, epithelium, and hemosiderin-laden macrophages. Moreover, endometriosis has been closely linked to infertility through such factors as chronic pelvic inflammation, ovarian endometriomas, impaired tubo-ovarian anatomy and function, reduced oocyte quality, and diminished endometrial receptivity. Concurrent conditions, such as adenomyosis further lower the success rate of assisted reproductive technologies.^4^,^8^

**Figure 1:**
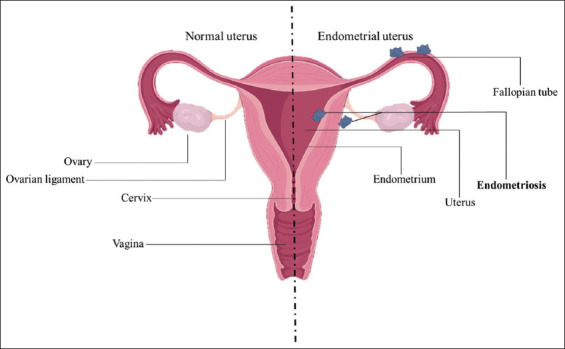
Representation of a normal uterus and an endometrial uterus. The diagram on the left illustrates a normal uterus with a healthy endometrial lining that thickens and sheds during the menstrual cycle. On the right side, the diagram shows the uterus with endometrial lesions on the walls of the uterus and fallopian tubes, which are characteristics of endometriosis. These lesions, composed of endometrium-like tissue, grow outside the uterus, leading to inflammation, pain, and potential infertility. The figure highlights how the ectopic growth of endometrial tissue in areas, such as the ovaries, fallopian tubes, and pelvic cavity can cause the development of endometriosis, disrupting normal reproductive function and contributing to the symptoms associated with the condition.

Endometriosis is mechanistically correlated with angiogenesis, lymphangiogenesis, and neurogenesis, which are all induced by inflammatory cells and lead to the ectopic growth of endometrial tissues. Mounting research evidence implies that endometriosis is a prevalent, chronic inflammatory condition that originates from the immune system. The peritoneal fluids associated with endometriosis contain various immune cells, including natural killer cells, neutrophils, mast cells, dendritic cells, and active macrophages. Nevertheless, the incapacity of these immune cells to recognize and eradicate ectopic endometrial cells suggests a disruption in their functioning.^9^ Figure 1 illustrates a uterus with an endometrial condition.

Despite intensive endometriosis research, there remains a need for a clearer understanding of its pathophysiology, diagnostic challenges, and complex relationship with fertility. This review article aimed to highlight the key aspects of the condition, including its prevalence, clinical presentation, and the present scientific insights into its causes and effects. By shedding light on the genetic, immunological, and inflammatory factors involved and the impact on reproductive health, this article sought to underscore the importance of continued research, improved diagnostic methods, and better management strategies for those affected by endometriosis. In addition, an extensive analysis was performed to offer deeper insight into the complex characteristics of this condition and its broad impact on women’s well-being.

## 2. Materials and methods

To accomplish this objective, an extensive literature search was conducted in various online databases, such as Scopus, ScienceDirect, PubMed, and ResearchGate. The search strategy incorporated specific keywords, including “endometriosis,” “genetic factors,” “stem cells,” “pathophysiology,” and “infertility,” to capture a wide and comprehensive range of relevant studies. Titles and abstracts of the retrieved articles were carefully screened to assess their suitability for inclusion in the review. The references of the chosen publications were further scrutinized to detect any additional key studies that might have been overlooked initially. This strategy aimed to gather a thorough and well-rounded body of research related to the interplay among endometriosis, genetic influences, stem cell involvement, underlying mechanisms, and reproductive challenges.

## 3. Pathophysiology of endometriosis

**Figure 2:**
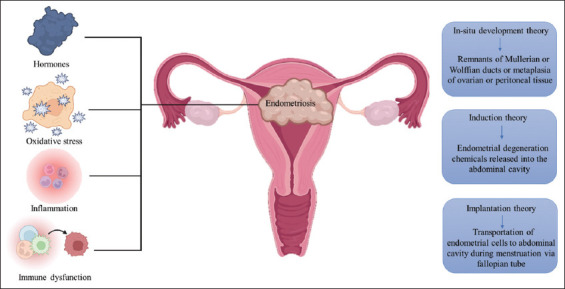
Pathophysiology of endometriosis. This diagram outlines the key factors contributing to the development and progression of endometriosis, including dysregulated hormone levels, oxidative stress, inflammation, and immune dysfunction. The pathogenesis is primarily based on three major theories: The *in situ* development theory, which suggests that endometrial tissue originates and grows in place within the pelvic cavity; the induction theory, which posits that endometrium-like cells are induced to grow in non-endometrial sites by environmental or genetic factors; and the implantation theory, which argues that endometrial cells from the uterus are displaced and implant in other areas of the body, leading to the formation of ectopic lesions.

The pathogenesis of endometriosis involves a wide array of factors, ranging from histopathological to anatomical ones. Based on its pathophysiological features, the American Fertility Society classifies endometriosis as deep and superficial endometriosis.^10^ Deep endometriosis, often presenting as a single nodule located in the vesicouterine fold or near the bowel, is categorized as adenomyosis externa, a significant cofactor in infertility.^11^ In contrast, superficial endometriosis is linked to pain at the vaginal introitus, which may result from provoked vestibulodynia or pelvic floor dysfunction.^12^ A key difference between deep and superficial endometriosis lies in their association with pain, with over 95% of pain being observed in deep endometriosis patients, against their superficial endometriosis counterparts. Factors associated with endometriosis include inflammation, oxidative stress, stem cells, hormones, genetic predisposition, immune dysfunction, and the upregulation of antiapoptotic factors 13. Several theories have been proposed to explicate the pathogenesis of endometriosis, offering insights into the initiation and progression of various endometrial lesions.^10^ Figure 2 shows the pathophysiology of endometriosis.

One of the primary hypotheses explaining the pathogenesis of endometriosis is the “*in situ* development” theory, which suggests that endometriosis originates at its specific ectopic location, potentially from remnants of the Müllerian or Wolffian ducts, or through metaplasia of ovarian or peritoneal tissues. Another important concept is the induction theory, which proposes that unidentified chemical signals released into the abdominal cavity during menstruation induce mesenchymal cells to differentiate into endometrium-like tissue. On the other hand, the transplantation or implantation theory, which is the most acknowledged hypothesis, suggests that, during retrograde menstruation, viable endometrial cells are transported, through the fallopian tubes, into the peritoneal cavity. These cells then adhere to the peritoneal lining or other pelvic structures, where they survive, proliferate, and form endometriotic lesions. This ectopic implantation of endometrial tissue is believed to be a pivotal mechanism in the growth of endometriosis, particularly in women with impaired immune clearance or other predisposing factors. Research strongly supports the decisive role of transplantation in the pathophysiology of endometriosis, highlighting its contribution to lesion formation and disease progression.^14^

In women affected by endometriosis, the retrogradely shed endometrial tissue often contains epigenetically altered stromal and epithelial cells, harboring mutations in cancer-associated genes, such as *PIK3CA* and *KRAS*. These altered fragments may implant on pelvic peritoneal surfaces or become trapped within ovarian cysts. Moreover, the action of estrogen and progesterone is facilitated through their respective nuclear receptors: Estrogen receptor and progesterone receptor, both being part of the nuclear/steroid receptor superfamily. In endometriosis, these receptors are often abnormally expressed due to epigenetic changes, especially in DNA methylation patterns. This dysregulation contributes to a hormonal imbalance, resulting in elevated estrogen levels, progesterone resistance, and persistent inflammation. A hallmark of endometriosis pathophysiology is this hormonal and inflammatory imbalance, which is further exacerbated by increased levels of prostaglandins and estrogen within the affected tissues.^15^ The induction theory further expands the concept of coelomic metaplasia by emphasizing the role of biochemical factors in the development of endometriotic lesions. It suggests that mesenchymal stem cells in the peritoneal cavity are chemically stimulated, possibly by substances shed from endometrial tissue, to transform into endometrium-like cells.^16^

In addition to these mechanisms, several other contributing factors have been proposed, including the volume and frequency of endometrial debris entering the peritoneal cavity, the efficiency of immune surveillance and clearance, and molecular abnormalities within the ectopic endometrial tissue. These elements are thought to influence the initiation and chronic progression of endometriosis synergistically.^17^

## 4. Factors affecting endometriosis

The development and progression of endometriosis are impelled by an intricate interplay among reproductive, hormonal, genetic, environmental, and lifestyle factors. Early menarche, short menstrual cycles, and heavy or sustained menstrual bleeding are the symptoms associated with a higher risk of endometriosis. These factors increase the likelihood of retrograde menstruation, a key mechanism implicated in the disease’s pathogenesis. Conversely, greater parity (having more pregnancies) appears to have a protective effect, potentially due to prolonged periods of anovulation and hormonal changes during pregnancy. The impact of oral contraceptive pills on endometriosis remains debated, though some studies suggest a potential protective effect through suppression of ovulation. Intrauterine device use has not shown a significant correlation with endometriosis risk. In addition, Müllerian anomalies, such as obstructive genital tract malformations, might influence the progression of endometriosis by impeding normal menstrual outflow and enhancing retrograde menstruation.^18^

Estrogen plays a central role in promoting endometrial tissue proliferation. Higher endogenous estrogen levels or exogenous estrogen exposure are associated with increased risk, while decreased estrogen production appears protective. Progesterone, on the other hand, exerts a protective effect by promoting the expression of anti-inflammatory and anti-proliferative genes. In women with endometriosis, progesterone resistance is commonly observed, leading to reduced responsiveness to its protective effects.^19^

Family studies have shown a strong hereditary component to endometriosis, with first-degree relatives having a significantly higher risk of developing the disease. GWAS have identified several susceptibility loci. Key genes associated with endometriosis include *WNT4 (*1p36.12), *VEZT* (12q22), and *GREB1 (*2p25.1), all of which are involved in hormone regulation, endometrial cell adhesion, and inflammatory response.^20^ These findings suggest that both genetic predisposition and epigenetic alterations contribute to disease risk and heterogeneity.

Interestingly, a higher body mass index (BMI) has been concomitant with a reduced risk of endometriosis. This inverse relationship is thought to result from more frequent anovulatory cycles and altered hormone levels in individuals with higher BMI. In contrast, taller stature has been linked to a slightly increased risk, although the mechanism remains unclear.^21^

Environmental exposures can influence endometriosis development. Cigarette smoke has been shown to disrupt steroid hormone biosynthesis and contains polycyclic aromatic hydrocarbons, heavy metals, and alkaloids, all of which may increase the risk of endometriosis by promoting inflammation and altering immune function. Alcohol and caffeine consumption have also been positively associated with increased risk, potentially due to their effects on estrogen metabolism and inflammatory pathways. In contrast, regular physical activity is protective, possibly due to its role in regulating hormones, reducing body fat, and enhancing immune surveillance.^22^

Specific food components have been linked to endometriosis risk. Elevated consumption of red meat and trans fats is correlated with a greater risk of developing the disease. A study found that women with high red meat consumption had a significantly greater risk of developing endometriosis, potentially due to increased estrogen levels. In contrast, diets rich in omega-3 fatty acids, fiber, green vegetables, and fruits, especially those with high antioxidant content, are associated with reduced risk. These foods are believed to exert anti-inflammatory effects and promote hormonal balance. In biological terms, fats may influence prostaglandin concentrations, potentially affecting ovarian function. Since exposure to unopposed estrogens is a known risk factor for endometriosis, diets rich in fat may contribute to higher circulating estrogen levels. Conversely, green vegetables and fruits provide micronutrients, such as Vitamin C, carotenoids, folic acid, and lycopene, which may offer protection by inhibiting abnormal cell proliferation. These dietary patterns are also consistent with reduced risks observed in other estrogen-related diseases, such as breast cancer, endometrial cancer, and uterine fibroids. Although epidemiological data on endometriosis and diet remain limited, the observed associations highlight the need for well-designed prospective studies to clarify these links.^23^

## 5. Genetic basis of endometriosis

The genetic basis of endometriosis involves a wide spectrum of gene variants and polymorphisms that influence various biological pathways, including inflammation, immune modulation, steroid hormone signaling, angiogenesis, and tissue remodeling. These genes are not only specific to endometriosis but also implicated in the pathophysiology of other chronic inflammatory and hormone-related disorders, indicating a shared molecular architecture.

One of the central mechanisms in endometriosis is the dysregulation of estrogen and progesterone receptor signaling, altering gene transcription and cellular responses in ectopic endometrial tissues. Estrogen receptor genes, *ESR1* and *ESR2*, encode receptors that mediate estrogen action. Polymorphisms in these genes can modulate receptor expression and sensitivity, influencing endometrial cell proliferation and resistance to apoptosis.^24^ *ESR2*, in particular, is overexpressed in endometriotic lesions, leading to a heightened estrogenic response that supports the subsistence and progression of ectopic endometrial tissue.

In addition, *TGFβ1* is another critical gene involved in the development of endometriosis. Under hypoxic conditions typical of ectopic lesions, *TGFβ1* fosters the expression of vascular endothelial growth factor, thereby engendering neo-angiogenesis, which is a hallmark of lesion progression.^25^ Polymorphisms in the *TGFβ* and *IL2RB* genes have been linked to altered immune responses, facilitating immune escape mechanisms that enable ectopic endometrial tissue to persist.

The human leukocyte antigen (HLA) system, particularly HLA class II alleles, has been associated with endometriosis, reflecting the autoimmune-like features of the disease. These alleles may impair immune surveillance, allowing ectopic cells to elude exposure and obliteration.^25^ Genes intricate in detoxification pathways, such as *GSTM1* and *GSTT1*, have also been studied. The null polymorphisms (complete deletion) in these genes lead to reduced detoxification capacity, increasing oxidative stress and inflammatory responses, potentially contributing to the onset and progression of endometriosis.^26^

Multiple single-nucleotide polymorphisms have been explored in GWAS. For example, *IL1A* (rs654209) is linked to increased pro-inflammatory cytokine production. It has been concomitant with a higher risk of endometriosis, particularly in advanced stages, though findings vary across studies. *NFE2L3* (rs12700667 and rs7798431), which encodes a protein that regulates oxidative stress response and transcriptional control, has been associated with disease susceptibility, albeit with inconsistent replication. In addition, *GREB1* (rs13394619), which encodes a protein responsive to estrogen receptor activation, is involved in the hormonal regulation of cell proliferation. Although initially associated with endometriosis, its significance remains inconclusive due to conflicting data. *FN1* (rs1250248), encoding fibronectin, facilitates the adherence and migration of cells. It has shown an association with advanced-stage endometriosis in some populations, although not all studies confirm this link. Meanwhile, *WNT4* (rs7521902) is one of the most consistently replicated loci and is involved in Wnt signaling, which is critical for Müllerian duct development and reproductive tract formation. Alterations in this pathway may predispose individuals to endometriosis by affecting tissue remodeling and cellular differentiation. *CDKN2B-AS1* (rs1537377) and *ID4* (rs7739264), associated with cell cycle control and differentiation, respectively, have shown limited or no reproducible association with endometriosis. Similarly, *RND3* (rs6734792), involved in cytoskeletal dynamics and cellular motility, also lacks consistent evidence of association. *VEZT* (rs10859871) is linked to cell adhesion and has shown positive association with endometriosis in several studies, suggesting a potential role in lesion attachment and implantation. Similarly, *ETAA1* (rs4141819), a gene involved in DNA damage response, was interrelated to an augmented risk of endometriosis in a single study; however, it requires further validation.^27^ Despite these advances, the reproducibility of GWAS findings remains a major challenge, with several associations failing to replicate across independent cohorts. This highlights the polygenic and heterogeneous nature of endometriosis and the need for larger, ethnically diverse studies to identify reliable genetic markers for diagnosis and therapeutic targeting.^27^,^28^

## 6. Action mechanism of stem cells in endometriosis

Adult stem cells preserve their undifferentiated nature through self-renewal while giving rise to various specialized cell types within their native tissues. These stem cells may be unipotent, producing only one specific cell type, or multipotent, capable of generating several cell types within a particular tissue. Unlike embryonic stem cells, which can differentiate into cell types from all three germ layers (endoderm, mesoderm, and ectoderm), adult stem cells exhibit a more limited, yet variable, differentiation potential.

**Figure 3:**
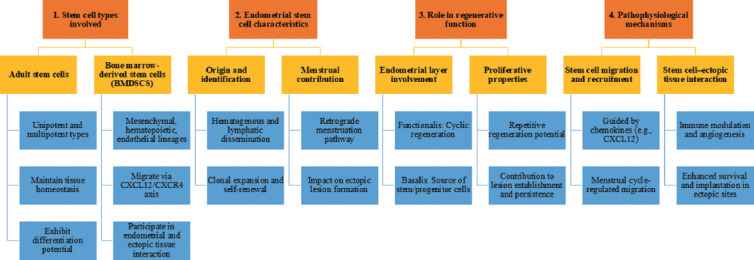
Structured representation of the roles and mechanisms of stem cells in the pathogenesis and progression of endometriosis. This figure illustrates the structured roles of various stem cell types in the development and maintenance of endometriosis. The framework is categorized into four main domains: (1) Stem cell types involved, including adult stem cells and bone marrow-derived stem cells with their key properties; (2) endometrial stem cell characteristics; (3) roles in regenerative function; and (4) pathophysiological mechanisms, highlighting stem cell recruitment guided by chemokines and interactions with ectopic tissues that promote immune modulation, angiogenesis, and lesion survival. This structured overview supports the hypothesis that stem cells significantly influence both the establishment and chronicity of endometriosis. Abbreviations: CXCL12: C–X–C motif chemokine ligand 12; CXCR4: C–X–C chemokine receptor type 4.

As adult stem cells begin to differentiate into tissue-specific cells known as progenitor cells, their capacity for self-renewal diminishes. Adult stem/progenitor cells are vital for preserving tissue equilibrium by replacing cells lost through regular cellular turnover or injury.^29^ Following the identification of a stem cell population in the endometrial tissue, present research has shifted toward the hypothesis that the migration of these cells through the bloodstream, lymphatic system, or retrograde menstruation may contribute to the initiation of endometriosis. The concept that endometriotic lesions may share clonal origins, with stem cells being shed during menstruation, supports the theory that endometrial stem cells play a crucial role in initiating endometriotic lesions. Figure 3 illustrates the roles and mechanisms by which stem cells contribute to the development and progression of endometriosis.

Stem cells originating from the bone marrow also seem to influence the disease’s pathophysiology. Since endometriosis may originate from multiple sources, such as the menstrual shedding of endometrial stem cells, the spread of endometrial or bone marrow-derived stem cells through blood or lymphatic circulation, and the retention of stem cells within Müllerian remnants, the involvement of stem cells presents a potential unifying factor among the various existing theories of its pathogenesis.^30^

### 6.1. Roles of stem cells in the endometrial regenerative function

The human endometrium is a distinctive tissue known for its remarkable ability to regenerate cyclically, driven by hormonal fluctuations throughout a woman’s reproductive lifespan. This regenerative capacity is primarily due to the presence of endometrial stem/progenitor cells located in the basalis layer, the permanent part of the endometrium that remains intact following menstruation. These stem cells replenish the cellular components of the functional layer, including luminal epithelial, stromal, glandular epithelial, and vascular endothelial cells, supporting tissue growth, differentiation, and repair.^31^,^32^

This regenerative phenomenon is not only critical for normal endometrial physiology but is also central to the pathogenesis of endometriosis. According to the stem cell theory of endometriosis, a subset of endometrial stem/progenitor cells, when shed during menstruation, may be retrogradely transported through the fallopian tubes into the peritoneal cavity. If these cells evade immune clearance and attach to ectopic sites, they can regenerate endometrium-like tissue outside the uterus, contributing to the formation and persistence of endometriotic lesions. This theory helps explain the recurrence of the disease and its resilience even after surgical removal of lesions. Moreover, these ectopic stem-like cells may exhibit altered gene expression profiles, dysregulated iron metabolism, and disrupted immunological signaling, contributing to chronic inflammation and lesion growth. Recent studies have shown that iron overload in the peritoneal environment, potentially due to repeated bleeding from endometriotic implants, can promote oxidative stress and influence stem cell behaviors. Adamyan *et al*.^33^ performed a systematic review and meta-analysis that highlighted the importance of iron metabolism markers within the peritoneal fluid of patients suffering from endometriosis, implying these markers may influence disease progression and sustained activity.

In addition, metabolomic profiling of endometriosis patients has revealed aberrant levels of specific metabolites that may influence the microenvironment and affect the proliferation and differentiation of stem cells intricate in lesion formation. The systematic review by Adamyan *et al*.^34^ on metabolomic biomarkers emphasizes the relevance of cellular metabolism in the etiology of endometriosis, further supporting the hypothesis that altered regenerative mechanisms contribute to disease pathogenesis. Thus, understanding the behavior and molecular regulation of endometrial stem/progenitor cells is essential for elucidating how ectopic implantation and cyclic regeneration of endometrium-like tissue occur in endometriosis. These insights may open novel avenues for targeted therapies aimed at modulating stem cell activity or blocking their ectopic establishment.

### 6.2. Roles of bone marrow-derived stem cells in the pathophysiology of endometriosis

Bone marrow-derived stem cells, including endothelial progenitor cells, hematopoietic stem cells, and bone marrow-derived mesenchymal stem cells (BMSCs), are believed to play a crucial role in the regeneration and pathology of the endometrium. These stem cells circulate in the bloodstream during the menstrual cycle, particularly in the proliferative phase, and may contribute to endometrial regeneration by differentiating into both stromal and epithelial cells. However, their involvement in the regeneration of uterine epithelial cells remains limited, suggesting that other factors influence their capacity to repair the endometrium effectively. One such critical factor is the hormonal environment, as the activity and fate of stem cells are affected by sex hormones, primarily estrogen and progesterone. Research indicates that the presence of these hormones enhances the recruitment of stem cells to endometrial tissues, both normal and ectopic, potentially contributing to the pathogenesis of endometriosis.^35^

The recruitment of stem cells to both eutopic and ectopic endometrial sites is thought to be regulated through various signaling pathways, with the C–X–C motif chemokine ligand 12 (CXCL12)/C–X–C chemokine receptor type 4 (CXCR4) axis being fundamental. This axis, which is well-known for its role in cancer metastasis, appears to govern the migration of stem cells to areas of endometrial tissue, where they can support vascularization and tissue remodeling. In endometriosis, this pathway is believed to facilitate the repositioning of BMSCs to ectopic sites, contributing to the formation of lesions. In addition, stem cells in endometriotic tissues express the chemokine receptor CXCR4 that can interact with its ligand, CXCL12, further promoting stem cell recruitment and potentially contributing to the growth and spread of ectopic endometrial lesions. The expression of CXCR4 is enhanced by hormonal stimuli, notably progesterone and estrogen, highlighting the complex interaction between stem cells, the immune system, and the hormonal milieu in endometriosis.

The immune system is also crucial in the recruitment and differentiation of these stem cells, as immune cells secrete cytokines and growth factors that influence stem cell activity. The inflammatory conditions that are often present in endometriosis can promote abnormal stem cell recruitment and differentiation, potentially contributing to the development of lesions and the chronic nature of the disease.^36^

**Figure 4:**
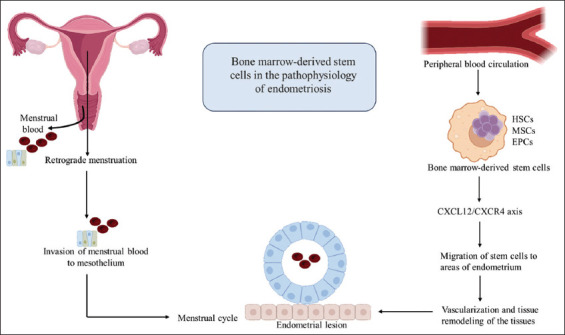
Bone marrow-derived stem cells in the pathophysiology of endometriosis. Bone marrow-derived stem cells contribute to the formation of endometrial lesions in endometriosis through the action of the CXCL12/CXCR4 axis, which facilitates the migration of stem cells to areas of the endometrium, ultimately leading to lesion formation. Abbreviations: CXCL12: C–X–C motif chemokine ligand 12; CXCR4: C–X–C chemokine receptor type 4; EPC: Endothelial progenitor cell; HSC: Hematopoietic stem cell; MSC: Mesenchymal stem cell.

In terms of therapeutic potential, targeting the conscription and differentiation pathways of BMSCs offers promising avenues for treatment. Modulating the CXCL12/CXCR4 axis or inhibiting the hormonal factors that enhance stem cell migration could help prevent the development of endometriosis or reduce the growth of ectopic lesions. In addition, stem cell-based interventions could hold promise for regenerative therapies to repair damaged endometrial tissue. By enhancing the regenerative capacity of the endometrium, stem cell-based therapies might offer a novel methodology for treating infertility and other complications associated with endometriosis. However, further research is required to better understand the precise mechanisms governing stem cell behavior in endometriosis and to develop targeted therapies that can modulate stem cell function without unintended side effects.^37^ Figure 4 depicts the role of BMSCs in the pathophysiology of endometriosis.

## 7. Endometriosis-associated complications

Endometriosis-associated complications include chronic pelvic pain, dysmenorrhea, dyspareunia, and infertility, all of which exert a significant negative influence on women’s quality of life. Despite achieving a long-awaited pregnancy, often through assisted reproductive technologies, individuals with endometriosis face an elevated susceptibility to encountering obstetric complications. These complications encompass, but are not restricted to, miscarriage, pre-term birth, pre-eclampsia, placental abnormalities, labor-related hemorrhage, delivery of small-for-gestational-age infants, stillbirth, and an increased likelihood of undergoing cesarean sections. Moreover, severe endometriosis complications during pregnancy can happen. One such consequence is spontaneous hemoperitoneum, a rare but potentially fatal illness that usually necessitates surgery.^38^ The complications associated with endometriosis and its risk factors are depicted in Table 1, while the genetic mutations linked to endometriosis and related cancers are shown in Table 2.

**Table 1:** The complications linked to endometriosis and their corresponding risk factors

Complication	Description	Risk factors	Mechanisms	References
Chronic pelvic pain	Persistent pain in the pelvic region, a hallmark symptom of endometriosis	Advanced stage of disease, deep infiltrating lesions	Inflammation, nerve infiltration, and pelvic adhesions	34,36,41
Infertility	Difficulty conceiving, especially in advanced stages of endometriosis	Pelvic adhesions, altered oocyte quality, and immune dysfunction	Distorted pelvic anatomy, hormonal imbalance, and inflammatory cytokines	35,37,39
Obstetric complications	Increased risk during pregnancy, including miscarriage and preterm birth	Pregnancy after assisted reproductive technologies, severe endometriosis	Placental abnormalities, hormonal imbalance, and inflammation	34,36,41
Endometriosis-associated ovarian cancer	An increased risk of developing certain types of ovarian cancer, including clear cell and endometrioid carcinoma	Long-standing endometriosis, genetic mutations (*e.g*., *ARID1A* and *PIK3CA*)	Oxidative stress, immune system dysregulation, and chronic inflammation	40,42,43
Hemoperitoneum	Spontaneous bleeding within the peritoneal cavity, sometimes requiring surgery	Severe endometriosis, particularly deep infiltrating forms	Ectopic tissue causing vessel rupture and bleeding	34, 41

**Table 2:** The genetic mutations associated with endometriosis and associated cancers

Gene	Associated condition	Function	Alteration type	Effects on endometriosis and cancer	References
*ARID1A*	Ovarian clear cell carcinoma	Tumor suppressor gene that regulates chromatin remodeling	Somatic loss-of-function mutation	Inactivation leads to cancer development and progression	40,42
*PIK3CA*	Ovarian clear cell carcinoma	Involved in cell growth, survival, and metabolism	Somatic activating mutation	Mutations increase tumorigenesis in endometriosis	40,42
*HOXA10*	Endometrial receptivity	Transcription factor critical for endometrial development and implantation	Altered gene expression (no mutation)	Downregulation impairs implantation, linked to infertility in endometriosis	38,39
*KRAS*	Endometrioid carcinoma	Regulates cell division and differentiation	Somatic activating mutation	Activating mutations contribute to tumorigenesis.	40,42
*PTEN*	Endometrioid carcinoma	Tumor suppressor, regulating the cell cycle and apoptosis	Somatic loss-of-function mutation	Loss of function increases cancer risk.	42

### 7.1. Endometriosis-associated infertility

The leading cause of infertility linked to endometriosis is significant pelvic anatomical distortion in the advanced stages of the disease, resulting in mechanical disruptions, such as pelvic adhesions. By decreasing egg release, impeding fertilization, producing disordered myometrial contractions, and changing sperm motility and embryo transit, these disruptions can interrupt fertility.

Advanced stages of endometriosis are more common in infertile women.^35^ Although the effects of severe illness on fertility are well established, it is still unclear how mild endometriosis influences fertility. The hypothesized causes include aberrantly expressed genes, growth and angiogenic agents, and inflammatory cytokines.^39^

Endometriosis affects the development of gametes and embryos by altering ovulation and oocyte production, with endometriomas and elevated inflammatory cells in the peritoneal fluid contributing to these effects. Furthermore, progesterone receptor dysregulation can cause luteal phase disruption, impacting endometrial receptivity. Peritoneal fluid inflammation can hurt sperm quality and function, while the inflammatory environment and heightened cytokine levels associated with endometriosis can influence the function and movement of the fallopian tube, affecting the transport of embryos. In addition, abnormal contractions in the myometrium related to endometriosis may impede the transportation of gametes and the implantation of embryos.^40^ Endometriosis is hypothesized to affect the eutopic endometrium, leading to implantation failure. The hypothesis suggests that abnormal gene expression, including genes, such as *Wnt7A*, in the endometrium of women affected by endometriosis could disrupt the typical epithelial-stromal polarity crucial for conception. The movement of cells between eutopic and ectopic endometrial tissue in both directions may contribute to implantation failure and alterations in gene expression.^41^

Several genes exhibit aberrant expression in endometriosis, including *CYP19A1*, which is involved in the synthesis of estrogen, and *Hoxa10*/*HOXA10*, which is crucial for endometrial receptivity. Implantation failure has been linked to dysregulation of progesterone receptors, progesterone resistance, and chronic production of matrix metalloproteinases, which break down extracellular matrices.^42^

### 7.2. Endometriosis-associated ovarian cancer

Recent studies employing whole-genome and targeted sequencing have identified frequent mutations in the *ARID1A* and *PIK3CA* genes in ovarian clear cell carcinomas, along with modest alterations in *PPP2R1A* and *KRAS*. Similar *PTEN*, *CTNNB1*, and *KRAS* mutations have been discovered in endometrioid cancer. Examining gene expression patterns indicates the inactivation of tumor suppressor genes *PTEN* and *ARID1A* in clear cells and endometrioid ovarian carcinomas, respectively. In addition, the oncogenic *KRAS* gene activation aligns with these observations.^43^

An important contributing element to the development of endometrioid and ovarian clear cell carcinomas is oxidative stress, which is implicated in several human illnesses. Endometriosis is associated with oxidative stress, resulting from recurrent episodes of bleeding, heme, and free iron within endometriotic lesions and endometrial tissue outside the uterus. Research indicates that endometriotic cysts have elevated stress-related variables and free iron levels compared to their non-endometriotic counterparts. When the generation of reactive oxygen species surpasses the capacity of cellular antioxidant defenses, oxidative stress-related damage results. Information derived from gene expression profiling indicates the involvement of oxidative stress in ovarian clear cell carcinoma, as reflected in the heightened expression of genes associated with redox processes. In clear cell carcinoma, there is an increase in the transcription factor hepatocyte nuclear factor 1-beta, which is associated with oxidative stress. Endometriosis, identified by both acute and chronic inflammation, is acknowledged as a pelvic inflammatory disease. Women affected by endometriosis exhibit elevated levels of cytokines and chemokines, along with a greater presence of activated macrophages.^44^

Inflammatory mediators and cytokines, including tumor necrosis factor-alpha (TNF-α), interleukin 6, and interleukin 1β, influence the development, expansion, and advancement of endometrioid ovarian cancer. Elevated TNF-α production is associated with ovarian cancer, impacting signaling pathways associated with cell adhesion, the cell cycle, angiogenesis, inflammation, and Notch signaling. Endometrioid and ovarian clear cell carcinomas are two gynecological cancers that are related to hyperestrogenism. Due to factors, such as elevated aromatase expression, endometriotic tissue lacks the enzymes necessary to convert estrogen into less powerful forms, raising local estrogen concentrations. By stimulating cytokines and producing prostaglandin E2, excess estrogen encourages cellular growth. Endometrioid subtypes are more likely to exhibit estrogen receptor and progesterone receptor than clear cell carcinomas, which typically lack them. In addition, DNA damage and mutations are more likely to occur in the endometriotic tissue microenvironment, characterized by proliferative pressure and enhanced reparative activity.^45^,^46^

## 8. Strategies for diagnosis

In recent years, molecular and genetic research development has created new opportunities to enhance endometriosis diagnosis. The discovery of potential biomarkers and genetic variations is one promising early diagnostic method. For example, changes in microRNA profiles and specific gene expressions have been proposed as potential biomarkers for early diagnosis. The time to diagnosis, which presently averages several years, could be significantly shortened by including these genetic markers in diagnostic testing. By integrating these indicators into clinical practice, healthcare professionals may identify individuals at risk for endometriosis earlier, enabling prompt intervention and improved disease management.^47^

Apart from genetic markers, the development of non-invasive diagnostic tools is another essential strategy to improve the diagnosis of endometriosis. While traditional methods, such as laparoscopy, are invasive and can be expensive and time-consuming, non-invasive techniques, including imaging technologies, blood-based tests, and liquid biopsy, offer the potential for earlier detection without the need for surgery.^48^ Liquid biopsy, which analyses circulating tumor DNA or RNA in blood samples, has gained attention for its ability to identify genetic alterations associated with endometriosis. These non-invasive methods could revolutionize diagnostic practices by providing a more accessible, affordable, and patient-friendly alternative to invasive procedures. As research in this area advances, it is crucial to validate these tools for clinical use to ensure their efficacy and reliability.^49^

Finally, one underutilized but crucial component of genetic insights into endometriosis is personalized treatment options. Clinicians can use genetic data to customize treatments for each patient according to their genetic composition and illness features. For example, some drugs or treatments, such as hormone therapies and targeted gene therapies, may be more effective in individuals with particular genetic mutations or molecular profiles. Targeting the genetic mechanisms linked to endometriosis, such as those involving angiogenesis, inflammation, or cell adhesion, may also provide new treatment alternatives. Incorporating genetic and molecular insights into clinical practice can lead to more effective management techniques and personalized medicines, eventually improving patient outcomes and quality of life.^50^

## 9. Conclusion

Endometriosis is a complex and multifaceted gynecological condition that profoundly impacts women’s health, particularly concerning fertility and quality of life. Despite significant advances in understanding its pathophysiology, challenges linger in diagnosing and managing this disease. The genetic basis of endometriosis, including the role of specific genes involved in inflammation, immune response, steroidogenesis, and DNA repair, continues to offer valuable insights into its development. Moreover, the involvement of stem cells from both bone marrow and the endometrium augments a layer of complexity to the perspective on the disease’s progression. Although substantial progress has been made, there is a need for innovative approaches to early detection and intervention, mainly through non-invasive diagnostic techniques and targeted therapeutics. Advancements in genomic and molecular research are pivotal for understanding the genetic underpinnings of endometriosis, ultimately uncovering specific gene expressions, mutations, and pathways linked to the disease. The burgeoning body of research also highlights the importance of preserving fertility in women with endometriosis, prompting further research into how the condition affects gametes and embryos. This evolving knowledge is poised to improve assisted reproductive technologies and treatment outcomes significantly. Overall, continual refinement of perspectives on endometriosis, including innovative approaches, such as the genetic-environmental theory, may enhance diagnostic accuracy, inform more effective treatment strategies, and ultimately improve the quality of life for affected women.

## Data Availability

Not applicable.
